# County-Level Sociodemographic Characteristics and Availability of COVID-19 Therapeutic Drugs

**DOI:** 10.1001/jamanetworkopen.2023.34763

**Published:** 2023-09-20

**Authors:** Alyssa Shishkov, Marcus R. Andrews, Sophie R. Alphonso, Yangyang Deng, Mohammad Moniruzzaman, Jonathan H. Cantor, Kosuke Tamura

**Affiliations:** 1 Socio-Spatial Determinants of Health (SSDH) Laboratory, Population and Community Health Sciences Branch, Division of Intramural Research, the National Institute on Minority Health and Health Disparities, the National Institutes of Health, Bethesda, Maryland; 2 Department of Health Behavior and Health Education, School of Public Health, University of Michigan, Ann Arbor; 3 RAND Corporation, Santa Monica, California

## Abstract

This cross-sectional study examines access to COVID-19 treatments in US counties by race and ethnicity, poverty rate, uninsured rate, and other population and community characteristics.

## Introduction

There continues to be a weekly mean of 14 500 COVID-19 cases and 250 deaths in the US, necessitating ongoing administration of therapeutic drugs.^1^ Research is limited on geospatial availability of COVID-19 treatments associated with local sociodemographic characteristics.^2^ Understanding treatment availability is critical; therefore, we investigated geospatial patterns of COVID-19 therapeutic drugs to assess possible sociodemographic-based disparities across US counties.

## Methods

Data on courses of COVID-19 therapeutic drugs available at different US health care sites on May 15, 2023, were obtained from the COVID-19 Public Therapeutic Locator.^3^ In accordance with the Common Rule, this cross-sectional study was exempt from review and informed consent because it used public data. We followed the STROBE reporting guideline.

Crude rate was expressed as the total number of COVID-19 therapeutic drug courses in each county and was standardized by dividing the number of courses by the total population of each county and multiplying by 100 000 population (eMethods in Supplement 1). We used global Moran I to assess unusually concentrated counties with high and low levels of treatments, which provided *z* scores with *P* values (eMethods in Supplement 1).^4,5^ Anselin^4^ local Moran I was then used to identify clusters of counties with available drugs that were different from neighboring counties. This analysis provided cluster types, including high-high clusters (counties with higher availability), low-low clusters (counties with lower availability), high-low and low-high outliers (counties with either high or low levels of availability surrounded by counties with either low or high levels of availability), and unclustered (counties with neither high nor low levels of availability).

Unpaired, 2-tailed *t* tests were used to compare sociodemographic characteristics (eg, self-identified race and ethnicity; poverty) inside and outside of clustered counties. Two-sided *P* < .05 indicated statistical significance. Analysis was performed with SAS 9.4 (SAS Institute Inc).

## Results

A total of 95 294 courses of COVID-19 therapeutic drugs were available, with a mean (SD) of 30.7 (91.8), in 3108 US counties. COVID-19 treatments were highly available (39.08-854.70 per 100 000 people) in some parts of New England, Kansas, and across the US (Figure, A). The Moran I value (0.09) for the total therapeutic drugs and *z* score (17.82) indicated that spatial clustering occurred across counties (*P* < .001). The Anselin local Moran analysis (Figure, B) showed high-high clusters around Maine, western Kansas and Nebraska, and eastern Montana. Low-low clusters appeared across the South and Midwest regions and some parts of the Western region.

**Figure.**
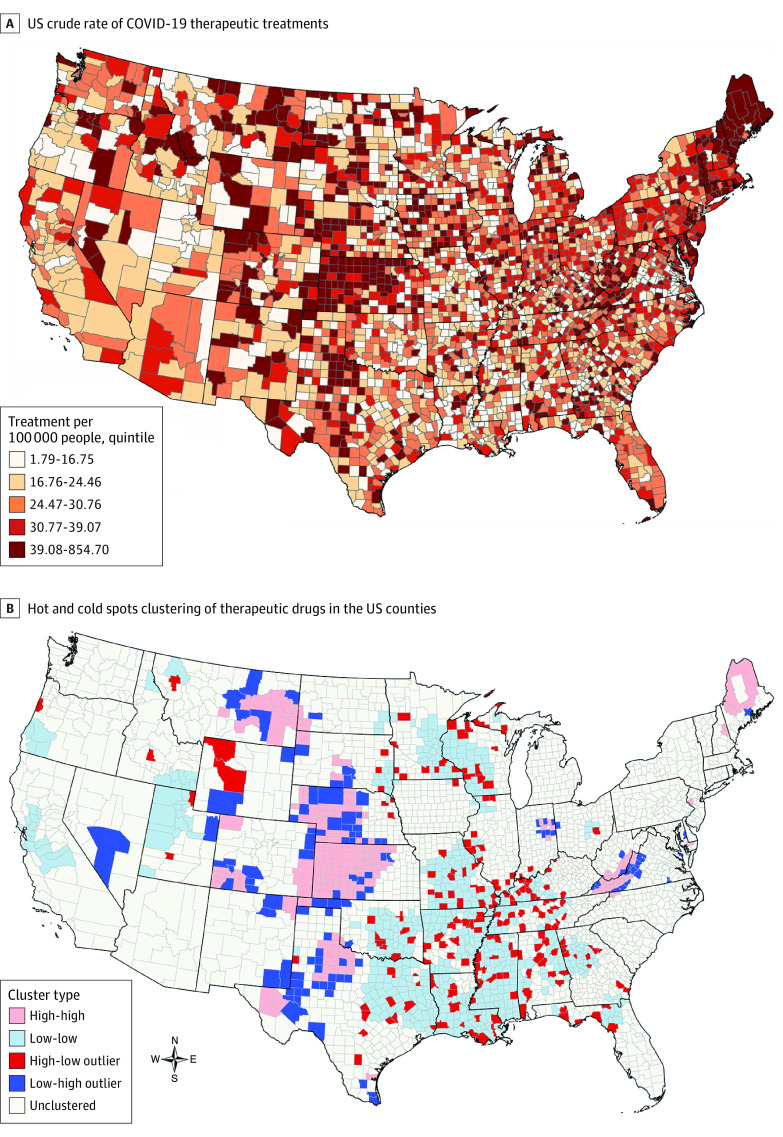
Crude Rate of COVID-19 Therapeutic Drugs and Clustering of Therapeutic Drugs in US Counties Data (panel B) were based on Anselin^4^ local Moran I values. Cluster types were high-high, indicating counties with high availability of COVID-19 therapeutic drugs; low-low, counties with low availability; high-low outlier, counties with high availability surrounded by counties with low availability; low-high outlier, counties with low availability surrounded by counties with high availability; and unclustered, counties with neither high nor low levels of availability.

High-high clusters vs unclustered counties had a lower percentage of Black population, COVID-19 crude rate, poverty rate, household income, Social Vulnerability Index (SVI), and COVID-19 Community Vulnerability Index (CCVI) (Table). Compared with unclustered counties, low-low clusters were more likely to have a higher percentage of Black population, poverty rate, uninsured rate, SVI, and CCVI and a lower percentage of Hispanic population and household income. High-low outliers were more likely than unclustered counties to have a higher percentage of Black population, COVID-19 crude rate, poverty rate, uninsured rate, SVI, and CCVI and a lower percentage of Hispanic population and household income. Low-high outlier clusters were more likely than unclustered counties to have a lower percentage of Black population, household income, and CCVI and a higher percentage of Hispanic population and uninsured rate.

**Table.** COVID-19 Therapeutic Drugs by County Cluster Characteristics

Characteristic	County cluster type, mean (SD), %				
	Unclustered^a^	Clusters		Outliers	
		High-high^a^	Low-low^a^	High-low^a^	Low-high^a^
All US counties, No./total No. (%)	2057/3108 (66.2)	182/3108 (5.9)	562/3108 (18.1)	195/3108 (6.3)	112/3108 (3.6)
Race and ethnicity^b^					
% Black or African American population^b^	7.73 (12.88)	1.92 (3.70)^c^	15.15 (18.72)^c^	14.14 (17.54)^c^	2.86 (6.34)^c^
% Hispanic or Latino population^b^	9.93 (14.23)	11.30 (14.90)	6.68 (8.28)^c^	5.97 (7.94)^c^	22.35 (25.71)^c^
Community characteristics					
COVID-19 crude rate per 100 000 people^d^	28 910.20 (8724.70)	27 672.80 (7720.40)^e^	29 601.10 (8938.50)	31 226.20 (6579.50)^c^	27 825.50 (7703.90)
Poverty rate	15.14 (6.31)	13.39 (6.02)^c^	17.43 (6.62)^c^	17.91 (6.01)^c^	15.60 (7.38)
Household income, US $	58 842.30 (14 907.10)	53 927.40 (9876.10)^c^	54 502.40 (14 658.00)^c^	53 233.60 (12 456.00)^c^	55 452.70 (10 513.20)^f^
Uninsured rate	9.48 (5.02)	10.13 (4.68)	11.14 (4.50)^c^	10.37 (4.34)^f^	12.84 (6.22)^c^
SVI^g^	0.48 (0.29)	0.36 (0.29)^c^	0.59 (0.28)^c^	0.59 (0.26)^c^	0.51 (0.29)
CCVI^h^	0.48 (0.28)	0.29 (0.28)^c^	0.61 (0.27)^c^	0.64 (0.26)^c^	0.41 (0.26)^f^

Abbreviations: CCVI, COVID-19 Community Vulnerability Index; SVI, Social Vulnerability Index.

^a^ Cluster types of counties: unclustered had neither high nor low levels of COVID-19 therapeutic drug availability; high-high clusters had high availability; low-low clusters had low availability; high-low outliers had high availability and were surrounded by counties with low availability; and low-high outliers had low availability and were surrounded by counties with high availability.

^b^ Race and ethnicity were self-identified. These data were obtained from the Agency for Toxic Substances and Disease Registry (https://www.atsdr.cdc.gov/placeandhealth/svi/data_documentation_download.html). We selected Black or African American and Hispanic or Latino groups for examination.

^c^ *P* < .001. Unpaired, 2-tailed *t* tests were used to compare values between unclustered and other cluster types.

^e^ *P* < .05.

^f^ *P* < .01.

^g^ The SVI consists of 16 social factors grouped into 4 themes and measures the relative social vulnerability of each county. The SVI ranges from 0 to 1, with a higher value indicating that a community is more vulnerable to external stressors such as human-driven or natural disasters or disease outbreaks.

^h^ The CCVI identifies communities more vulnerable to COVID-19 using data from the SVI and the Centers for Disease Control and Prevention. The CCVI ranges from 0 to 1, with a higher value indicating that a community is more vulnerable to socioeconomic, environmental, and health care risks from the COVID-19 pandemic.

## Discussion

The results showed sociodemographic-based disparities in geographic clustering of COVID-19 therapeutic drugs, highlighting disparities in access to these drugs. As reflected in the literature, counties with high poverty and uninsured rates and individuals with high SVI and CCVI had significantly lower access to COVID-19 therapeutic drugs.^6^ Moreover, counties with high proportions of Black individuals had significantly lower access to COVID-19 treatments.^6^ With the end of the COVID-19 Public Health Emergency, these results highlight an important gap in treatment access. A study limitation was that size of area-level data could have differential results.^5^ Finer geographic scale, such as Census tracts, should be used for future investigations.

## Notes

Supplement 1.
eMethods.

Supplement 2.
Data Sharing Statement
