# Supplementary material for: County-Level Sociodemographic Characteristics and Availability of COVID-19 Therapeutic Drugs
Source: JAMA Netw Open. 2023 Sep 20;6(9):e2334763. doi: 10.1001/jamanetworkopen.2023.34763 (PMC10512099; doi:10.1001/jamanetworkopen.2023.34763)
Supplement: Supplement 1. — eMethods. [file jamanetwopen-e2334763-s001.pdf]

Supplemental Online Content

Shishkov A, Andrews MR, Alphonso SR, et al. County-level sociodemographic characteristics and availability of COVID-19 therapeutic drugs. *JAMA Netw Open*. 2023;6(9):e2334763. doi:10.1001/jamanetworkopen.2023.34763

eMethods

This supplemental material has been provided by the authors to give readers additional information about their work.

© 2023 Shishkov A et al. *JAMA Network Open*

eMethods

Data on available courses of COVID-19 therapeutic drugs (Paxlovid, Renal Paxlovid, Lagevrio) as of May 15, 2023, was obtained from the COVID-19 Public Therapeutic Locator.[1] Because mapping using spatial analysis was only possible across contiguous areas, Alaska, Hawaii, and U.S. territories were removed, resulting in a total of 3,108 U.S. counties used for the analyses. The total number of drugs available in each county was standardized by dividing the total number of courses by the population of each county and multiplying by 100,000.
Statistical analysis was conducted using Global Moran's I, and Anselin Local Moran's I. Global Moran's I value ranging from -1 to +1 was used to determine if the proportion of available COVID-19 drug courses were spatially autocorrelated at the county level (i.e., the extent to which the availability of drug courses in one area have the similar availability in close areas).[2] A positive Global Moran's I value indicates that the proportion of COVID-19 drug courses is clustered across counties.[3] In contrast, a negative value indicates that the proportion of COVID-19 therapeutic drugs is dispersed across the counties.

A Moran's I value of 0.05 was obtained for the U.S. counties, indicating that spatial clustering occurred across the U.S. counties. Furthermore, a z-score of 17.82 was obtained, indicating that the results were statistically significant and that the likelihood that the clustering occurred by chance is less than 1%.

Anselin Local Moran's I was conducted to identify counties with high or low COVID-19 drug proportions that are statistically different from surrounding counties.[3]

Table 1 in the manuscript presents the number of counties clustered together based on the Local Moran's I. Each sociodemographic characteristic within high- and low-clustered counties was compared to corresponding values in unclustered counties using a t-test with p-values.

Social Vulnerability Index (SVI) included 16 social factors grouped into four themes used to determine the relative social vulnerability of each county.

COVID-19 Community Vulnerability Index (CCVI) is an index used to determine communities more vulnerable to COVID-19 using data from the SVI and the CDC.

The spatial clustering analysis is more informative than a traditional statistical analysis because the results indicate actual locations with unusually high and low availability of treatments. As a next step, comparing the clusters (e.g., High-High clusters vs. unclustered) provides valuable information about the identified clusters.

eReferences
1. HealthData.gov. COVID-19 Public Therapeutic Locator. Accessed May 15, 2023, https://healthdata.gov/Health/COVID-19-Public-Therapeutic-Locator/rxn6-qnx8/data
2. Cliff AD, Ord K. *Spatial autocorrelation: A review of existing and new measures with applications*. U. of Bristol; 1969.
3. Andrews MR, Tamura K, Best JN, et al. Spatial Clustering of County-Level COVID-19 Rates in the U.S. *Int J Environ Res Public Health*. Nov 19 2021;18(22)doi:10.3390/ijerph182212170

© 2023 Shishkov A et al. *JAMA Network Open*
