# Supplementary material for: County-Level Sociodemographic Characteristics and Availability of COVID-19 Therapeutic Drugs
Source: JAMA Netw Open. 2023 Sep 20;6(9):e2334763. doi: 10.1001/jamanetworkopen.2023.34763 (PMC10512099; doi:10.1001/jamanetworkopen.2023.34763)
Supplement: Supplement 2. — Data Sharing Statement [file jamanetwopen-e2334763-s002.pdf]

Data Sharing Statement

Shishkov. County-Level Sociodemographic Characteristics and Availability of COVID-19 Therapeutic Drugs. *JAMA Netw Open*. Published September 20, 2023. doi:10.1001/jamanetworkopen.2023.34763

Data
Data available: No

Additional Information
Explanation for why data not available: The data are publicly available, and the link to the dataset was included in the manuscript.
